# The Antigerminative Activity of Twenty-Seven Monoterpenes

**DOI:** 10.3390/molecules15096630

**Published:** 2010-09-21

**Authors:** Laura De Martino, Emilia Mancini, Luiz Fernando Rolim de Almeida, Vincenzo De Feo

**Affiliations:** 1 Dipartimento di Scienze Farmaceutiche, Università degli Studi di Salerno, via Ponte Don Melillo, 84084 Fisciano (Salerno), Italy; E-Mails: ldemartino@unisa.it (L.D.M.); emancini@unisa.it (E.M.); 2 Departamento de Botânica, Instituto de Biociências de Botucatu, UNESP - Campu de Botucatu Distrito de Rubião Júnior, S/N, 18.618-000, Botucatu-SP, Brazil; E-Mail: rolimdealmeidalf@yahoo.com.br (L.F.R.d.A.)

**Keywords:** monoterpenes, germination, radicle elongation, phytotoxicity

## Abstract

Monoterpenes, the main constituents of essential oils, are known for their many biological activities. The present work studied the potential biological activity of twenty-seven monoterpenes, including monoterpene hydrocarbons and oxygenated ones, against seed germination and subsequent primary radicle growth of *Raphanus sativus* L. (radish) and *Lepidium sativum* L. (garden cress), under laboratory conditions. The compounds, belonging to different chemical classes, showed different potency in affecting both parameters evaluated. The assayed compounds demonstrated a good inhibitory activity in a dose-dependent way. In general, radish seed is more sensitive than garden cress and its germination appeares more inhibited by alcohols; at the highest concentration tested, the more active substances were geraniol, borneol, (±)-β-citronellol and α-terpineol. Geraniol and carvone inhibited, in a significant way, the germination of garden cress, at the highest concentration tested. Radicle elongation of two test species was inhibited mainly by alcohols and ketones. Carvone inhibited the radicle elongation of both seeds, at almost all concentrations assayed, while 1,8-cineole inhibited their radicle elongation at the lowest concentrations (10^−5^ M, 10^−6^ M).

## 1. Introduction

Environmental constraints of crop production systems have stimulated interest in alternative weed management strategies. In fact, the use of synthetic herbicides may threaten sustainable agricultural production and has resulted in serious environmental problems, such as the enhanced incidence of resistance in weeds to important herbicides and the increased environmental pollution and health hazards [1]. Therefore, there is a need for new herbicides less harmful for mammalian health and environment. In recent years, there is an increasing interest in the development of natural products as bio-herbicides. The plants offer a remarkable potential for selective biological weed management and starting point for the development of biodegradable compounds [2] through the production and release of substances from the leaves, flowers, seeds, stems, and roots of living or decomposing plant materials. Inhibition of plant growth by other plants in their neighborhood has been known for a long time and the possible use of natural compounds in weed management has been well documented [3,4]. Under appropriate conditions, plant metabolites may be released in quantities suppressive to developing weed seedlings [5]. A variety of natural products have been identified, including essential oils, that inhibit seed germination and plant growth [6]. Terpenes are the largest group of secondary products, the monoterpenes being the simplest representatives. They are ubiquitous in higher plants and algae [7] and these compounds are often found in copious amounts compartmentalized in glandular hairs (trichomes) of the plant surface [8]. It has been demonstrated that terpenes are involved in multiple ecological functions in plants, such as protection against herbivores and microbial diseases, attraction of pollinators, and in allelopathy [8,9]. Many monoterpenes have been described as potent inhibitors of seed germination and growth of several plant species [10]. Although a relatively large number of highly phytotoxic substances are derived from the terpenoid pathway, the mode of action of few of these phytotoxins is well understood [11].

Recently, we studied the phytotoxicity of twelve essential oils from Mediterranean plants, reporting also their chemical features [12]. For these reasons, the aim of this work was to study single monoterpenoids, main constituents of previous cited essential oils, in order to evaluate their biological activity and their chemical features, towards germination and radicle elongation of selected seeds. The assayed compounds belonged to different chemical groups, e.g., alcohols, phenols, aldehydes, ketones, acetates, hydrocarbons and ethers. We tested the potential phytotoxic activity of each compound on seed germination and growth of the primary radicle elongation of *Raphanus sativus* L. (radish) and *Lepidium sativum* L. (garden cress).

## 2. Results and Discussion

In the present study, the biological inhibitory activity of twenty-seven commercial monoterpenes, including oxygenated monoterpenes and monoterpene hydrocarbons, were tested on seed germination and radicle elongation of radish and garden cress. The tested monoterpenoids have shown, in a dose-dependent way, an effective inhibitory activity on the two studied seeds (Table 1 and Table 2).

Generally, the germination of radish seeds is more affected by monoterpenes than garden cress seeds. In particular, at 10^−3^ M, a rather high concentration in the field of natural compounds, the substances provoked, in a significant way, inhibition on germination with this order of potency: geraniol > carvone > borneol > β-citronellol > α-terpineol > camphor > menthol > menthone > limonene > citral. Carvone and geraniol affected the germination of radish seeds with a 96% inhibition. Menthone, limonene, carvone and camphor also inhibited, in a significant way, the germination of these seeds at 10^−4^ M. At the lowest doses assayed, the radish seeds were significantly inhibited only by 1,8-cineole. The germination of garden cress was less sensitive to inhibition by monoterpenes. In particular, geraniol, carvone and limonene affected the germination of these seeds with an inhibition of 58%, 34% and 20%, respectively. At the other concentrations tested, no monoterpenes inhibited garden cress germination in a significant way. In the matter of radicle elongation, monoterpenes affected both seeds, in a similar way. At 10^−3^ M, carvone, borneol, limonene and camphor were the most active compounds in inhibition of radish seedling growth, provoking an inhibition from 79% (carvone) to 44% (camphor). Carvone, limonene and *p*-cymene inhibited the radicle elongation of garden cress also at 10^−4^ M. At the lowest concentrations tested, unless 1,8-cineole and citronellol, the radicle elongation values are not significantly different from control. At 10^−3^ M, borneol, thymol, carvacrol, citronellol and camphor affected the radicle growth of garden cress, in a significant way: in particular, borneol inhibited the radicle elongation of seeds with a 86% inhibition (at 10^−3^ M) and with a 40% inhibition (at 10^−5^ M). Carvone inhibited significantly the seedling growth of these seeds both at 10^−3^ M and 10^−4^ M; also at 10^−6^ M, this compound provoked a 36% inhibition of seedling growth. As well as for the radish, at the lowest concentrations, 1,8-cineole inhibited, in a significant way, the primary root growth.

Alcohols (borneol, citronellol, geraniol, α-terpineol) appeared as the most inhibitory compounds (10^−3^ M), followed by ketones (carvone, menthone, camphor) and aldehydes, against germination of tested seeds. Alcohols and ketones were the most inhibitory on both radish and garden cress radicle growth. On the whole, different degrees of inhibition were observed when compared with control groups, whereas oxygenated monoterpenes have more potent herbicidal effects on seed germination as compared with monoterpene hydrocarbons. As shown in Table 1 and Table 2, monoterpene hydrocarbons have less or no phytotoxic effects on the seed germination of two seeds. Moreover, alcohol derivatives of oxygenated monoterpenes were more phytotoxic than their acetate derivatives. In some cases, monoterpenes did not affect radicle elongation of two seeds assayed, but they inhibited only seed germination: in fact, geraniol, α-terpineol, menthone, menthol, citral, at the highest concentrations tested, inhibited significantly germination of seeds. On the other hand, carvacrol, thymol, *p*-cymene and 1,8-cineole inhibited the seedling growth of the seeds, but they did not affect their germination.

Ploszynski and co-workers [13] reported the phytotoxic effects of triazine herbicides on *Lepidium sativum* seeds: these substances have been assayed at rather high concentrations comparables to our compounds; also 2,4-dimethylamine derivatives showed a strong effect on radicle growth of the same seeds [14]. Other studies reported the herbicide effects of different substances, in relatively high amounts, against radish seed [15]. 

Previous studies showed that essential oils isolated from various plant species, on the whole, and specifically monoterpenes, exert potent herbicidal effects on weed germination and primary root growth of several other species [4,10,16,17,18,19]. In agreement with our results, several Authors [10,18] previously reported that 1,8-cineole and camphor have strong phytotoxic effects against various plant species; citronellal, citronellol, linalool [20,21], α-pinene [10,21] and limonene [10] are known as high inhibitors of seed germination and seedling growth. In several papers, monoterpene hydrocarbons showed to posses lower inhibitory activity than oxygenated compounds [16,18,20]. 

In this study, some oxygenated monoterpenes showed high inhibitory activity on germination and radicle elongation of radish and garden cress seeds: it is well known that these compounds have phytotoxic effects that may cause anatomical and physiological changes in seedlings: reduction in some organelles such as mitochondria, accumulation of lipid globules in the cytoplasm, may be due to inhibition of DNA synthesis or disruption of membranes [17]. Batish and coworkers [22] reported that the oil from *E. citriodora* inhibited root growth by suppressing of mitotic activity. Singh and coworkers [23] reported that the oil of *Artemisia scoparia* inhibited germination and plant root growth through generation of ROS-induced oxidative stress. Kordali and coworkers [18] reported thatβ-citronellol, nerol and terpinen-4-ol completely inhibited seed germination and seedling growth of tested plants. Similar results were found in our study. Moreover, our data showed that the tested alcohols, phenols and ketones were more active than others classes. 

## 3. Experimental Section 

### 3.1. Monoterpenes

Twenty-seven commercial monoterpenes were purchased from Sigma-Aldrich Co. (Milan, Italy). They included representative phenols, alcohols, acetates, ethers, hydrocarbons, ketones and aldehydes. All compounds were used as received without further purification. The compounds tested were: (±)-β-citronellol, (±)-citronellal, (-)-α-pinene, (-)-β-pinene, α-terpinene, γ-terpinene, α-terpineol, 1,8-cineole, citral, thymol, carvacrol, α+β-thujone, camphene, (±)-camphor, (-)-borneol, *p*-cymene, myrcene, menthone, (±)-menthol, geraniol, geranyl acetate, linalool, linalyl acetate, (*R*)-(-)-α-phellandrene, estragole, (*R*)-(-)-carvon, limonene. The purity of the compounds, as reported by the supplier, was checked by GC analysis. 

### 3.2. Biological Assay

A bioassay based on radish germination and subsequent radicle growth was used to study phytotoxic effects of the twenty-seven compounds on seeds of *Raphanus sativus* L. *cv.* “Saxa” (radish) and *Lepidium sativum* L. (garden cress). The seeds of *Lepidium sativum* L. and *Raphanus sativus* L. were purchased from Blumen srl, Piacenza, Italy. The seeds were surface-sterilized in 95% ethanol for 15 s and sown in Petri dishes (Ø = 90 mm), containing five layers of Whatman filter paper, impregnated with 7 mL of distilled water (control) or 7 mL of tested solution of the essential oil at the different assayed doses. The germination conditions were as follow: 20 ± 1°C, with natural photoperiod. The twenty-seven standard, dissolved in water–acetone mixture (99.5:0.5), were assayed at the concentrations of 10^−3^ M, 10^−4^ M, 10^−5^ M and 10^−6^ M. Controls performed with water-acetone mixture alone showed no appreciable difference in comparison with controls in water alone. Seed germination process was observed directly in Petri dishes, each 24 hours. Seed was considered germinated when the protrusion of the radical became evident [24]. After 120 hours (on the fifth day), radicle length were measured in centimeters. Each determination was repeated three times, using Petri dishes containing 15 seeds each. Data are expressed as the mean ± SEM of both germination and radicle length.

### 3.3. Statistical analysis

Data were ordered in homogeneous sets, and the Student’s *t* test of independence was applied [25].

## 4. Conclusions

In general, the effects of an essential oil on seed germination and seedling growth is often explained in terms of the individual effects of some main constituents: an essential oil is a mixture of many compounds in different proportions, and it is often not known whether and how they might interact [16]. We examined a large number of individual compounds belonging to all major groups of monoterpenoids in an attempt to identify structural features, responsible for the activity expressed on germination and subsequent seeding growth, which would consequently allow prediction of the level of inhibition. 

Our data demonstrated that the tested monoterpenes showed phytotoxic activity and these compounds could be used both as potential bio-herbicides and as lead structures for the development of new, potentially safe and ecocompatible pesticides. Further studies will be needed to investigate costs, selectivity, safety and mode of action of these monoterpenes.

## Figures and Tables

**Table 1:** Effects of different concentrations of monoterpenes on radish and garden cress germination, 120 hours after sowing. The data are expressed as the mean of three replicates ± SE.

	*Raphanus sativus* Germinated seed				*Lepidium sativum* Germinated seed			
	[10^−3^] M	[10^−4^] M	[10^−5^] M	[10^−6^] M	[10^−3^] M	[10^−4^] M	[10^−5^] M	[10^−6^] M
Control	12.4 ± 1.4	12.4 ± 1.4	12.4 ± 1.4	12.4 ± 1.4	14.4 ± 0.5	14.4 ± 0.5	14.4 ± 0.5	14.4 ± 0.5
(-)-Borneol	2.0 ± 0.0***	12.5 ± 2.1	10.5 ± 2.1	10.0 ± 0.0	13.5 ± 0.7	13.5 ± 0.7	13.5 ± 0.7	14.0 ± 1.4
(±)-Camphor	6.0 ± 1.4**	9.0 ± 1.4*	13.0 ± 0.0	13.5 ± 2.1	13.0 ± 1.4*	13.5 ± 0.7	12.5 ± 0.7**	15.0 ± 0.0
(±)-Citronellal	11.0 ± 1.4	13.0 ± 1.4	10.5 ± 0.7	11.5 ± 0.7	15.0 ± 0.0	15.0 ± 0.0	14.0 ± 1.4	14.5 ± 0.7
(±)-Menthol	6.5 ± 2.1**	11.0 ± 2.8	11.5 ± 2.1	11.0 ± 4.2	14.0 ± 1.4	14.5 ± 0.7	13.5 ± 2.1	13.5 ± 0.7
(±)-β-Citronellol	3.5 ± 2.1***	13.0 ± 1.4	10.0 ± 1.4	10.5 ± 0.7	13.0 ± 1.4	15.0 ± 0.0	14.0 ± 1.4	15.0 ± 0.0
(R)-(-)Carvone	0.5 ± 0.7***	8.0 ± 2.8*	9.0 ± 2.8	10.5 ± 0.7	9.5 ± 2.1***	14.5 ± 0.7	13.5 ± 0.7	14.5 ± 0.7
1,8-Cineole	11.0 ± 2.8	10.5 ± 0.7	12.5 ± 0.7	9.5 ± 0.7	14.0 ± 1.4	13.5 ± 0.7	14.0 ± 1.4	14.0 ± 1.4
Camphene	13.5 ± 2.1	11.5 ± 0.7	11.5 ± 2.1	10.0 ± 2.8	13.5 ± 0.7	13.0 ± 1.4	13.5 ± 0.7	13.5 ± 0.7
Carvacrol	10.5 ± 0.7	13.0 ± 1.4	12.5 ± 2.1	11.5 ± 3.5	13.5 ± 0.7	14.0 ± 0.0	15.0 ± 0.0	14.0 ± 1.4
Citral	8.0 ± 1.4*	12.5 ± 0.7	11.0 ± 1.4	11.5 ± 0.7	15.0 ± 0.0	14.5 ± 0.7	15.0 ± 0.0	15.0 ± 0.0
Estragole	11.5 ± 2.1	12.5 ± 0.7	12.0 ± 2.8	12.5 ± 0.7	15.0 ± 0.0	14.0 ± 1.4	15.0 ± 0.0	12.5 ± 2.1
Geraniol	0.5 ± 0.7***	10.0 ± 0.0	12.5 ± 0.7	9.5 ± 0.7	6.0 ± 1.4***	15.0 ± 0.0	15.0 ± 0.0	14.5 ± 0.7
Geranyl acetate	9.5 ± 2.1	11.0 ± 0.0	11.5 ± 0.7	10.5 ± 0.7	13.5 ± 0.7	14.5 ± 0.7	14.5 ± 0.7	13.5 ± 2.1
Limonene	7.0 ± 1.4**	8.0 ± 2.8*	8.5 ± 4.9	11.5 ± 2.1	11.5 ± 3.5*	15.0 ± 0.0	14.5 ± 0.7	13.0 ± 1.4*
Linalool	10.0 ± 1.4	10.5 ± 2.1	9.5 ± 2.1	12.0 ± 2.8	14.0 ± 0.0	13.5 ± 0.7	13.0 ± 1.4	14.5 ± 0.7
Linalyl acetate	11.0 ± 1.4	12.5 ± 0.7	11.5 ± 3.5	13.0 ± 0.0	15.0 ± 0.0	14.0 ± 0.0	15.0 ± 0.0	14.0 ± 1.4
Menthone	6.5 ± 2.1**	7.0 ± 1.4**	11.0 ± 1.4	10.0 ± 0.0	13.5 ± 0.7	14.0 ± 1.4	13.5 ± 0.7	13.5 ± 2.1
Myrcene	14.0 ± 0.0	14.0 ± 1.4	14.0 ± 1.4	13.0 ± 1.4	14.5 ± 0.7	14.5 ± 0.7	14.5 ± 0.7	14.0 ± 0.0
*p*-Cymene	13.0 ± 1.4	11.5 ± 2.1	10.5 ± 2.1	13.0 ± 2.8	13.5 ± 0.7	13.5 ± 2.1	14.0 ± 0.0	14.0 ± 0.0
Thymol	10.5 ± 0.7	12.0 ± 1.4	13.5 ± 0.7	11.5 ± 2.1	7.0 ± 1.4	15.0 ± 0.0	15.0 ± 0.0	15.0 ± 0.0
α-Phellandrene	13.0 ± 0.0	11.5 ± 2.1	12.5 ± 0.7	11.0 ± 0.0	14.0 ± 0.0	15.0 ± 0.0	14.5 ± 0.7	15.0 ± 0.0
α-Pinene	12.5 ± 0.7	11.5 ± 0.7	13.0 ± 1.4	13.0 ± 1.4	14.5 ± 0.7	15.0 ± 0.0	14.5 ± 0.7	15.0 ± 0.0
α-Terpinene	13.0 ± 0.0	13.0 ± 0.0	12.0 ± 0.0	13.0 ± 2.8	15.0 ± 0.0	14.5 ± 0.7	14.5 ± 0.7	15.0 ± 0.0
α-Terpineol	5.0 ± 0.0***	14.0 ± 0.0	13.5 ± 2.1	12.5 ± 2.1	14.5 ± 0.7	15.0 ± 0.0	15.0 ± 0.0	15.0 ± 0.0
α-β Thujone	10.0 ± 0.0	12.0 ± 1.4	12.0 ± 1.4	12.0 ± 0.0	15.0 ± 0.0	15.0 ± 0.0	15.0 ± 0.0	15.0 ± 0.0
β-Pinene	12.0 ± 0.0	11.0 ± 0.0	13.0 ± 1.4	13.5 ± 2.1	15.0 ± 0.0	15.0 ± 0.0	15.0 ± 0.0	15.0 ± 0.0
γ-Terpinene	14.0 ± 0.0	14.5 ± 0.7	14.0 ± 1.4	11.0 ± 1.4	14.5 ± 0.7	14.5 ± 0.7	14.5 ± 0.7	15.0 ± 0.0

The values followed by * (* p < 0.05; ** p < 0.01; *** p < 0.001), are statistically different according to the Student’s *t* test.

**Table 2:** Effects of different concentrations of monoterpenes on radish and garden cress radical elongation, 120 hours after sowing. The data are expressed in cm as the mean of three replicates ± SE.

	*Raphanus sativus* Radicle length				*Lepidium sativum* Radicle length			
	[10^−3^] M	[10^−4^] M	[10^−5^] M	[10^−6^] M	[10^−3^] M	[10^−4^] M	[10^−5^] M	[10^−6^] M
Control	1.8 ± 0.4	1.8 ± 0.4	1.8 ± 0.4	1.8 ± 0.4	1.9 ± 0.2	1.9 ± 0.2	1.9 ± 0.2	1.9 ± 0.2
(-)-Borneol	0.5 ± 0.1**	1.9 ± 1.1	1.6 ± 1.1	1.6 ± 0.8	0.2 ± 0.1***	1.6 ± 0.7	1.1 ± 0.6*	1.3 ± 0.7
(±)-Camphor	1.0 ± 0.5*	1.2 ± 0.8	2.1 ± 0.9	1.6 ± 1.0	1.1 ± 0.5**	1.5 ± 0.6	1.7 ± 0.7	1.6 ± 0.9
(±)-Citronellal	1.7 ± 1.4	2.0 ± 1.2	1.5 ± 0.8	1.8 ± 1.1	1.8 ± 0.7	2.0 ± 0.7	2.0 ± 0.7	2.1 ± 0.6
(±)-Menthol	1.5 ± 0.8	1.7 ± 1.0	1.9 ± 0.8	1.9 ± 0.7	1.4 ± 0.6	1.8 ± 0.9	1.9 ± 0.7	1.6 ± 1.0
(±)-β-Citronellol	1.1 ± 0.8	1.4 ± 0.7	1.4 ± 0.7	1.5 ± 0.9	0.7 ± 0.5***	1.7 ± 0.6	1.6 ± 0.8	2.0 ± 1.2
(R)-(-)Carvone	0.1 ± 0.1***	0.9 ± 0.5**	1.4 ± 0.8	1.0 ± 0.5**	0.4 ± 0.2***	0.8 ± 0.4***	1.2 ± 0.7	1.3 ± 0.6*
1,8-Cineole	1.2 ± 0.7	1.1 ± 0.8	0.9 ± 0.6**	0.9 ± 0.5**	1.8 ± 1.0	1.5 ± 0.8	1.2 ± 0.6*	1.2 ± 0.5**
Camphene	1.2 ± 0.8	1.5 ± 0.7	1.6 ± 1.2	1.6 ± 0.8	1.9 ± 0.9	1.7 ± 0.8	2.1 ± 0.9	1.3 ± 0.7
Carvacrol	1.8 ± 1.0	2.0 ± 1.3	2.0 ± 1.3	2.2 ± 1.6	0.6 ± 0.3***	1.7 ± 0.8	1.8 ± 0.8	2.1 ± 0.7
Citral	1.3 ± 0.8	2.1 ± 1.1	2.3 ± 1.3	1.3 ± 0.4*	1.4 ± 0.6	1.9 ± 0.7	1.7 ± 0.9	1.7 ± 0.7
Estragole	1.3 ± 0.8	1.3 ± 0.9	1.4 ± 0.9	1.3 ± 0.7	1.3 ± 0.9	1.3 ± 0.6	1.6 ± 0.8	1.5 ± 1.0
Geraniol	0.3 ± 0.5	1.7 ± 0.9	1.8 ± 0.9	1.4 ± 0.7	0.8 ± 0.4	1.6 ± 0.8	1.5 ± 0.7	2.1 ± 0.6
Geranyl acetate	1.2 ± 0.4	1.3 ± 0.5	1.4 ± 0.8	1.4 ± 0.8	1.5 ± 0.7	1.6 ± 0.8	1.3 ± 0.7	1.8 ± 0.7
Limonene	0.9 ± 0.4**	0.9 ± 0.5**	1.9 ± 1.1	1.4 ± 0.7	1.8 ± 0.8	1.3 ± 0.9	1.5 ± 0.6	1.2 ± 0.8
Linalool	1.4 ± 1.1	1.5 ± 0.7	1.2 ± 0.7	1.4 ± 0.6	1.6 ± 0.7	2.0 ± 0.9	1.4 ± 0.7	1.7 ± 0.8
Linalyl acetate	1.1 ± 0.6	1.6 ± 1.0	1.6 ± 0.8	1.5 ± 0.7	1.7 ± 0.6	1.4 ± 0.6	1.9 ± 0.7	1.4 ± 0.6
Menthone	1.2 ± 0.6	1.4 ± 0.6	1.4 ± 0.7	1.6 ± 0.8	1.4 ± 0.8	1.6 ± 0.8	2.4 ± 0.9	1.8 ± 1.0
Myrcene	2.5 ± 1.6	2.4 ± 1.8	1.9 ± 1.2	1.7 ± 1.2	1.8 ± 0.6	2.0 ± 0.8	1.6 ± 0.6	2.0 ± 0.6
*p*-Cymene	1.7 ± 0.9	1.1 ± 0.6*	1.3 ± 0.6	1.5 ± 0.8	2.3 ± 0.8	1.7 ± 0.7	1.9 ± 0.9	2.6 ± 1.1
Thymol	1.5 ± 0.9	2.1 ± 1.3	2.5 ± 1.4	1.7 ± 0.9	0.5 ± 0.1***	1.9 ± 1.1	2.1 ± 0.7	1.9 ± 0.8
α-Phellandrene	1.7 ± 0.8	2.0 ± 1.2	1.4 ± 0.8	1.4 ± 0.8	1.8 ± 1.0	1.8 ± 1.0	2.0 ± 0.8	2.2 ± 0.8
α-Pinene	1.4 ± 0.9	1.5 ± 0.8	1.7 ± 0.7	1.4 ± 0.8	1.6 ± 0.6	1.6 ± 0.7	1.6 ± 0.6	2.2 ± 0.9
α-Terpinene	1.5 ± 0.6	1.9 ± 1.4	1.7 ± 1.4	1.9 ± 1.3	1.9 ± 0.8	2.0 ± 0.6	1.8 ± 0.7	2.0 ± 1.0
α-Terpineol	2.1 ± 1.0	2.3 ± 1.3	2.0 ± 1.7	2.0 ± 1.2	1.7 ± 0.5	2.2 ± 0.5	2.2 ± 0.6	1.7 ± 0.7
α-β Thujone	1.5 ± 1.3	1.8 ± 1.1	1.6 ± 1.2	1.7 ± 0.9	1.7 ± 0.6	1.9 ± 0.9	1.8 ± 0.9	2.2 ± 0.9
β-Pinene	1.3 ± 0.6	1.5 ± 0.9	1.3 ± 0.6	1.4 ± 0.8	2.0 ± 0.8	2.0 ± 0.8	1.7 ± 0.8	1.8 ± 0.8
γ-Terpinene	1.7 ± 1.2	2.2 ± 1.3	1.7 ± 1.3	1.9 ± 1.4	1.6 ± 0.5	1.9 ± 0.9	1.7 ± 0.5	2.0 ± 0.8

The values followed by * (* p < 0.05; ** p < 0.01; *** p < 0.001), are statistically different according to the Student’s *t* test.
